# Patient satisfaction after lower limb replantation surgery for traumatic amputation - a qualitative study

**DOI:** 10.1186/s12891-023-07076-4

**Published:** 2024-01-02

**Authors:** Yantao Pei, Yakubu Ibrahim, Gang Wang, Yuliang Sun, Benjamin Tze Keong Ding, Qingjia Xu

**Affiliations:** 1 Department of Hand and Foot Surgery, Department of Orthopedic Surgery, Qilu Hospital of Shandong University, 107 Wen Hua Xi Lu 107, Jinan, 250012 China; 2 Department of Orthopaedic Surgery, Woodlands Health, Singapore, Singapore

**Keywords:** Traumatic lower limb amputation, Replantation Surgery, Life satisfaction, Patient perspective, Physical function

## Abstract

**Background:**

The majority of published literature clinically assesses surgical outcomes after lower limb replantation for traumatic amputations. However, patients’ satisfaction and quality of life may not be accurately measured through rigid scoring using standardized patient reported outcome measures.

**Purpose:**

The aim of this study was to qualitatively assess patient satisfaction and factors associated with achieving good outcomes after successful lower limb replantation surgery.

**Methods:**

A semi-structured interview was conducted with 12 patients who underwent lower limb replantation surgery following traumatic amputation injuries. The interview focused on the patients’ experience and satisfaction throughout their injury, surgical journey, rehabilitation and reintegration into their communities. An inductive and deductive thematic analysis was applied using the recorded transcripts to evaluate the overall satisfaction of the patients after lower limb replantation surgery.

**Results:**

The following observations emerged from the structured themes among all the patients interviewed: (1) Family and social support was significantly associated with improved qualities of life and satisfaction after lower limb replantation; (2) Patients were generally satisfied with their outcomes despite limitations in physical capabilities; (3) Satisfaction was associated with acceptance of their cosmetic deformity; (4) Social integration and being able to participate in a meaningful manner was associated with greater satisfaction after recovery.

**Conclusions:**

Patients who undergo lower limb replantation can have a significantly improved quality of life if they have strong social support, are able to contribute in a meaningful manner to their communities after surgery, and are accepting of their cosmetic deficiencies.

## Introduction

Patients with traumatic lower limb amputation suffer debilitating physical and psychological repercussions. Managing these injuries can be arduous, requiring protracted rehabilitation and posing a challenge for functional integration into activities of daily living. [1]Despite the decrease in amputation rates, an estimated 1.6 million Americans live with limb loss: 53% due to nontraumatic lower extremity amputation, 45% due to trauma, and 2% due to cancer. [1] Lower-extremity amputation, have a major negative impact on the health and well-being of those who are affected, and hospitalizations for these injuries continue to pose a significant healthcare and economic burden. Annually, the medical costs of caring for Medicare individuals who had nontraumatic amputations totaled $4.3 billion [2].

The lower limb replantation procedure may be indicated for specific cases. It is considered for complete or partial amputations with viable limbs, especially when the injury mechanism, such as trauma, allows for successful reconstruction of blood vessels, nerves, and tissues [3, 4]. The patient’s overall health, age, lifestyle, and psychological readiness play pivotal roles in the decision-making process. Nevertheless, the decision to perform lower limb replantation should be individualized, considering the patient’s unique circumstances and the expertise of the medical team [4, 5].

The current study reaffirms the value of efforts to better understand how patient’s perception of outcome can differ from those of surgeons in various therapeutic settings [1, 2].

This study aimed to evaluate patient satisfaction following lower limb replantation and lengthening procedures and to investigate the effect of patients’ sociodemographic factors, injury patterns, and treatment decision-making on satisfaction after complex limb reconstruction surgery.

## Materials and methods

The institutional review board, Research Ethics Committee of Qilu Hospital, Shandong University (Approved number KYLL-2020(KS)-573), approved this study. Between 2020 and 2021, we conducted semi-structured interviews with 12 patients (10 males and 2 females) who had suffered traumatic lower limb amputations and subsequently underwent limb replantation surgeries. Patients who were 18 years or older and had a lower limb replantation surgery at a minimum of 6 months before interview were included in the study.

Patients with irreversible ischemia, extensive soft tissue loss, medical comorbidities, inadequate vascular structures, advanced age, and previous failed replantation were excluded from the replantation procedure.

All the patients suffered trauma-related lower extremity amputation (LLA). The mean age was 44.5 years (range 20–60 years). The time duration between the trauma and the arrival at the hospital was four to six hours. Three out of twelve patients had amputation at the level of the ankle joint and nine patients had below the knee amputation (BKA). All patients received initial treatment at a local clinic prior to transfer to our facility for definitive treatment.

A purposeful sampling strategy based on patient demographics was used. Face-to-face interviews were scheduled and conducted by three trained qualitative interviewers who were not involved in the care of the patients. The durations of the interviews lasted between 45 min and one and a half hours.

The interview guide was structured to understand individual patient’s experience from the time of injury until the time of interview. This entailed questions focusing on changes in their physical activity limitations, psychological and emotional endurance, and social and family ties during recovery.

The interview draft was analyzed by the study team which included a psychologist and orthopedic surgeons. The final codebook was reviewed by a separate team and the codes organized into themes. A QDA Miner Lite software (Provalis Research, Montreal, Canada) was used for data analysis.

**Fig. 1:**
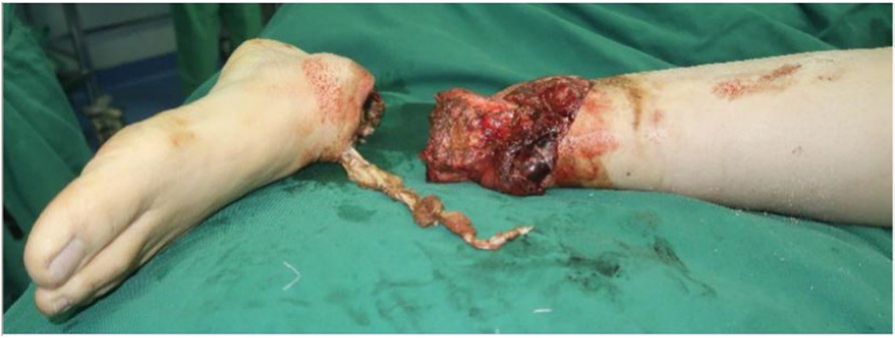
Left tibiotalar ankle joint dislocation

A 36-year-old man suffered a traumatic injury while operating a machine, resulting in a complete amputation of the lower extremity at the tibiotalar ankle joint. The wound was contaminated with soil and exhibited horizontal skin exfoliation, exposing the articular surface (Fig. 1).

He received initial bandaging at a local clinic and was then transferred to our facility 4 h after the injury. He arrived in a stable hemodynamic condition and was promptly scheduled for debridement and replantation of the severed limb. Under general anesthesia, two surgical teams were assembled- one working on the stump, and the other on the amputated part of the limb.

**Fig. 2:**
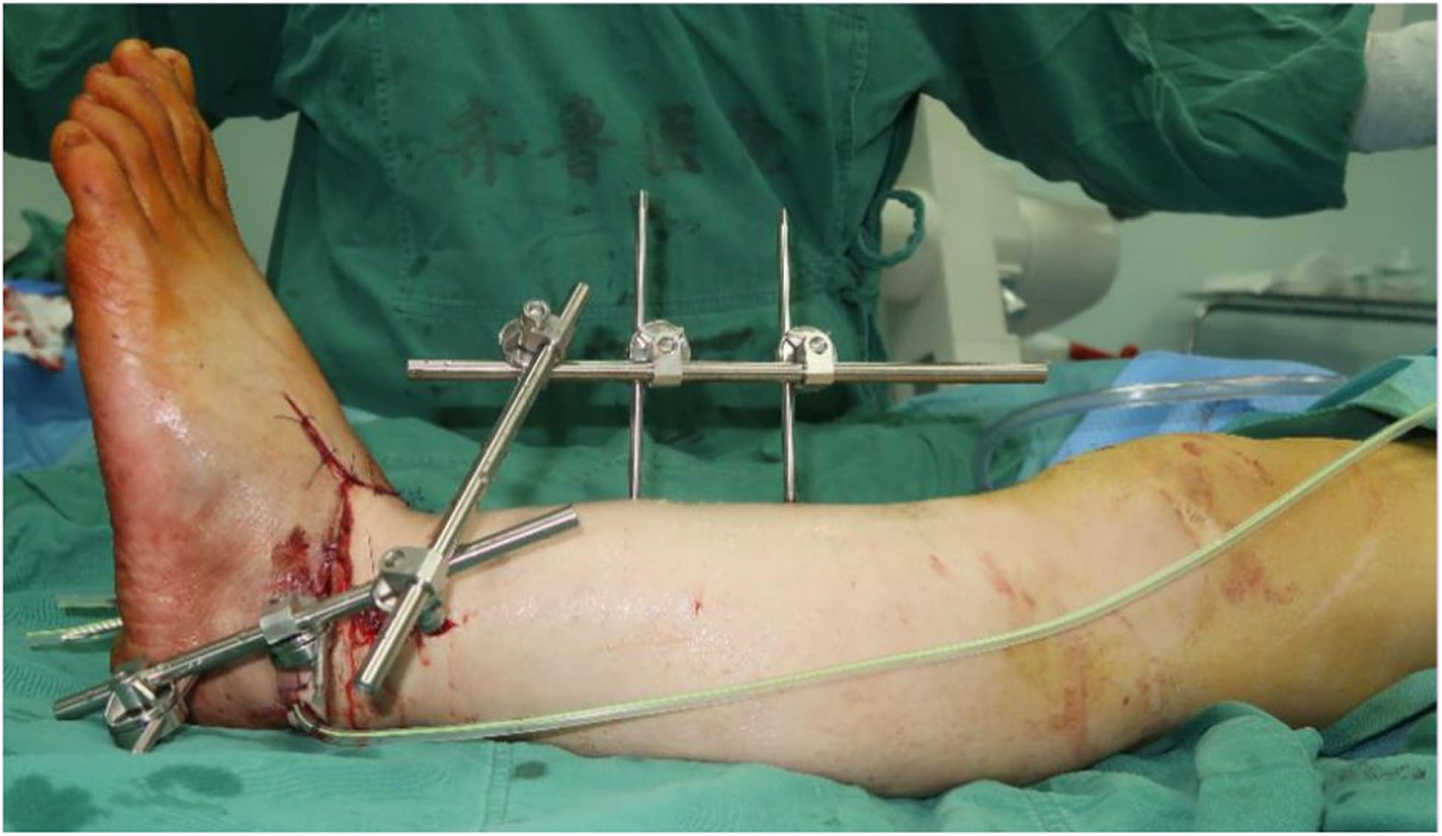
Shortened ankle arthrodesis, stabilization of ankle joint by inserting Kirschner wire through the calcaneus to the tibia bone with an external frame in place

**Fig. 3:**
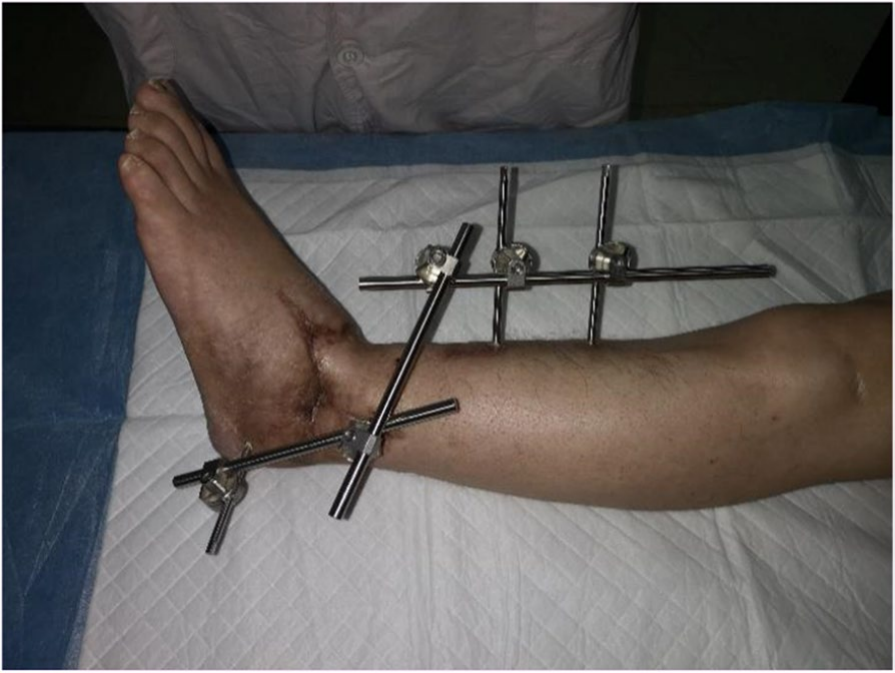
Condition of wound healing after 10 months

We conducted a thorough wound debridement and removed any foreign bodies that had penetrated the wound. Fractures were detected in both the medial and lateral malleoli. We explored and identified the distal and proximal dorsal pedis arteries, the posterior tibial artery, their corresponding veins, the tibial nerve, sural nerve, and superficial and deep peroneal nerves. To prepare for ankle arthrodesis, we shortened the distal tibia and stabilized the ankle joint by inserting a Kirscher wire from the calcaneus to the tibia, using an external frame (Fig. 2). Severed tendons and tissues were repaired. We performed end-to-end anastomosis of the posterior tibia, dorsalis pedis artery, and its accompanying vanae comitantes, along with the saphenous vein. Subsequently, we repaired the severed nerve tissues. The entire replantation and revascularization procedure too 6 h from the time of injury. When the tourniquet was loosened, there was adequate blood supply to the toes and the capillary refill time was satisfactory. The intraoperative and postoperative course was uneventful, and the wound healed without complications (Fig. 3).

**Fig. 4:**
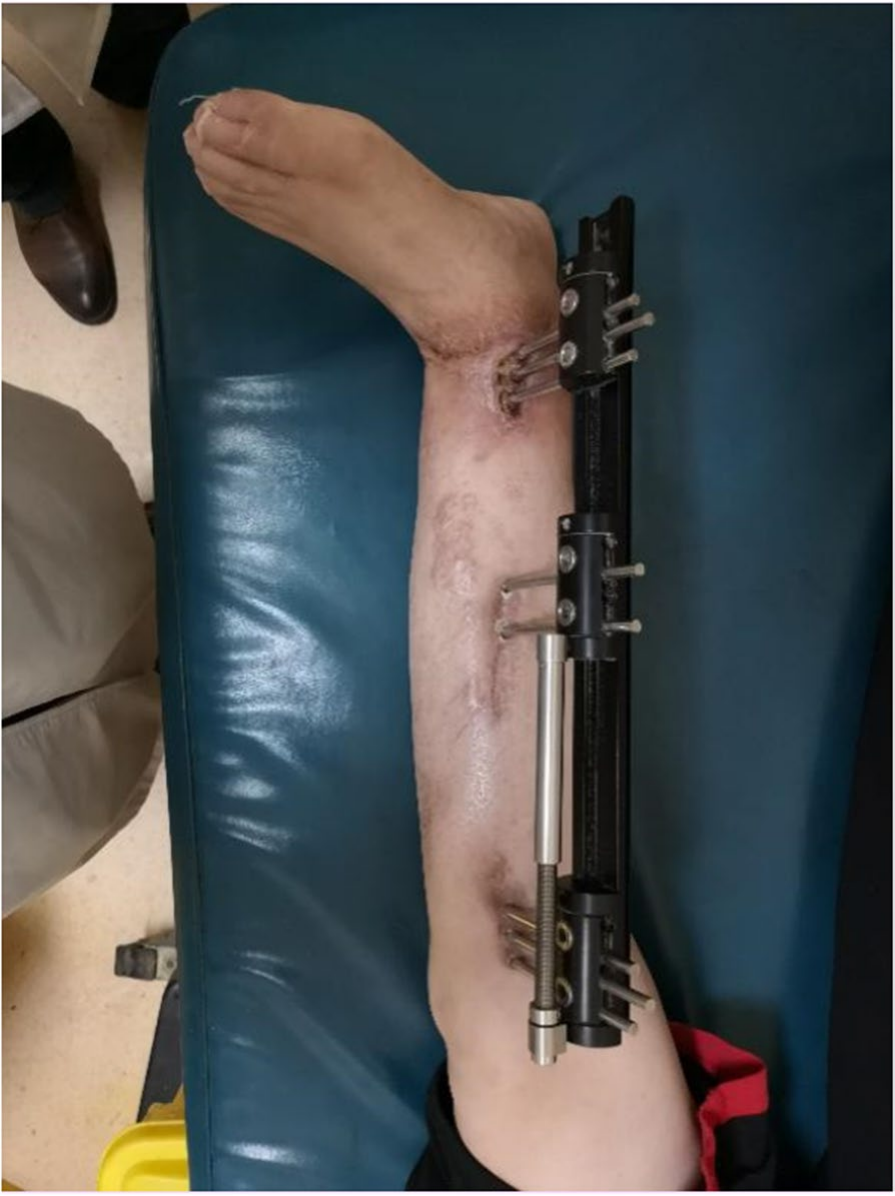
Conditions after left tibial osteotomy and lengthening external fixation

One year after the primary procedure, the patient presented for further treatment due to significant shortening deformity of the left limb that had been operated on. Examination revealed evident scar tissue at the surgical site, limited ankle joint range of motion, and decreased sensation in the left foot. The patient underwent a left tibial osteotomy and lengthening with external fixation. Under general anesthesia, we performed a minimally invasive osteotomy approximately 1 cm below the tibial tuberosity. Rods were extended to create the desired distraction. Intraoperative fluoroscopy confirmed a satisfactory osteotomy position and distraction (Fig. 4).

### Case 2

**Fig. 5:**
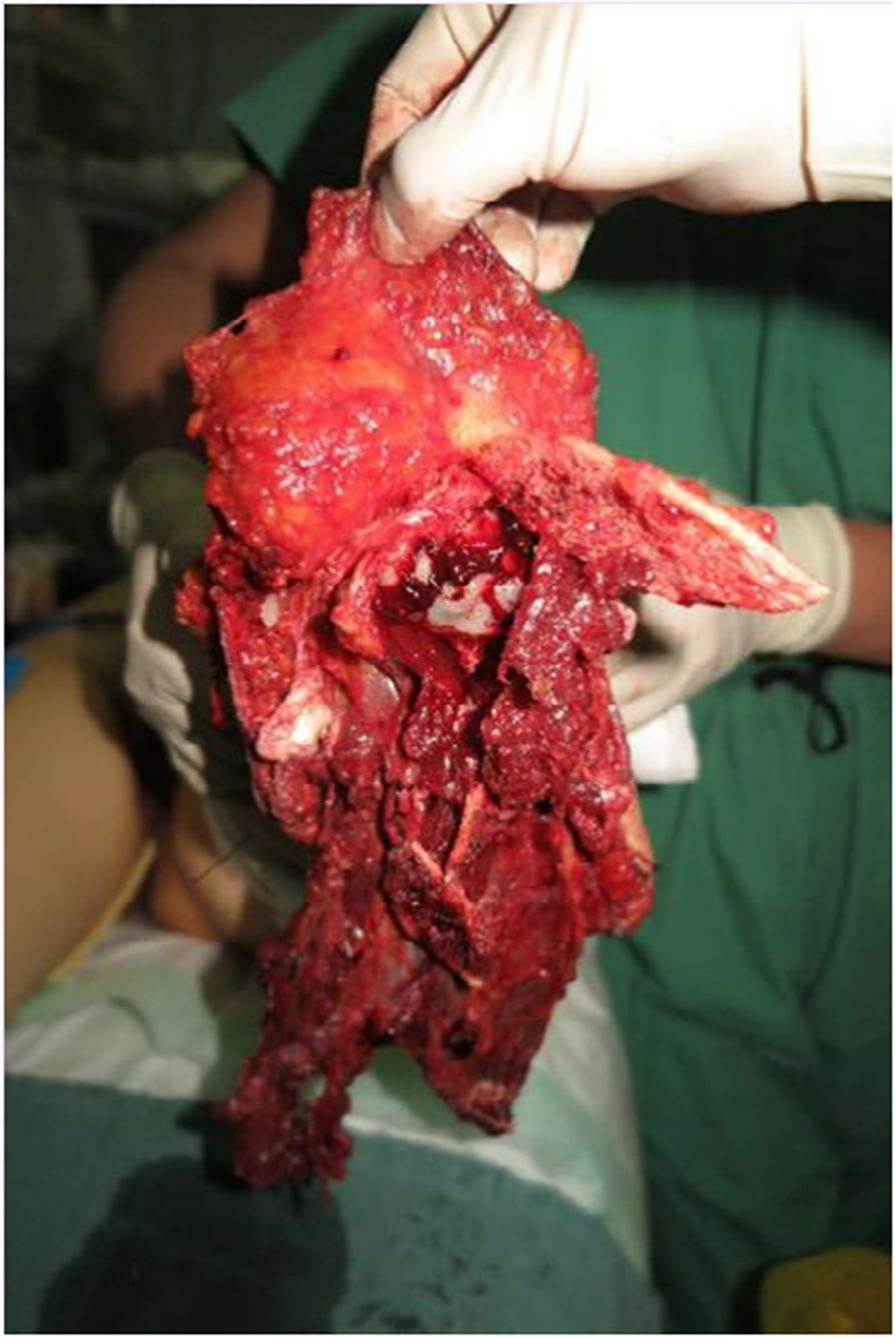
Middle and upper part of the left leg with comminuted fractures of the tibia and fibula

A 50-year-old individual was involved in a motor vehicle accident and suffered a left leg amputation. The patient received a bandaging and hemostasis treatment at a local hospital before being transferred to our hospital for further treatment, which occurred 5 h after the incident. Severe trauma was evident in the middle and upper part of the left leg, including comminuted fractures of the tibia and fibula. Additionally, the proximal fibula was missing (Fig. 5).

**Fig. 6:**
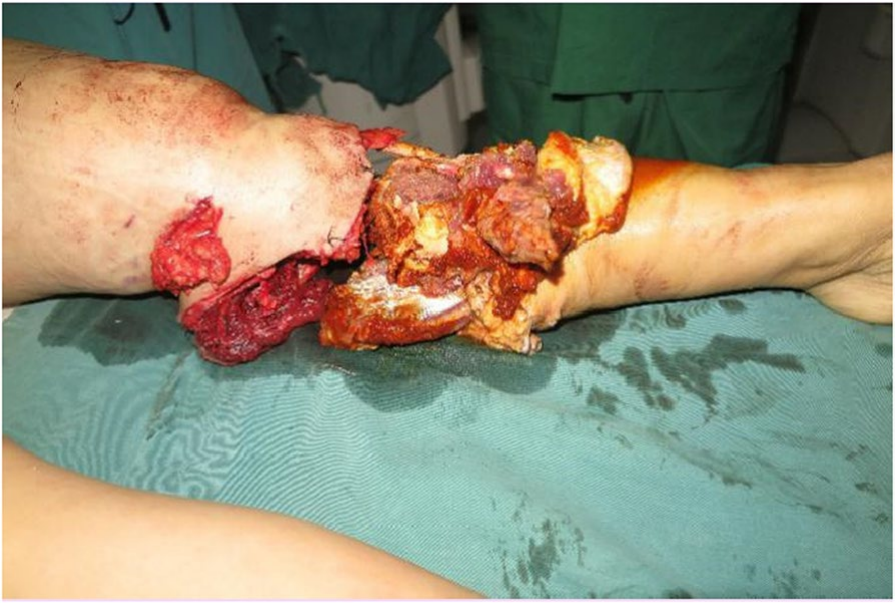
Stabilization of shaft and proximal tibia and fibula with K-wire and external fixator

Under general anesthesia, we employed a two-team approach to work on the severed leg and the stump simultaneously. Wound debridement was performed, and blood stasis and foreign bodies were removed. We fixed the comminuted fractures of the shaft and proximal tibia and fibula with K-wire and external fixator to ensure stabilization (Fig. 6).

**Fig. 7:**
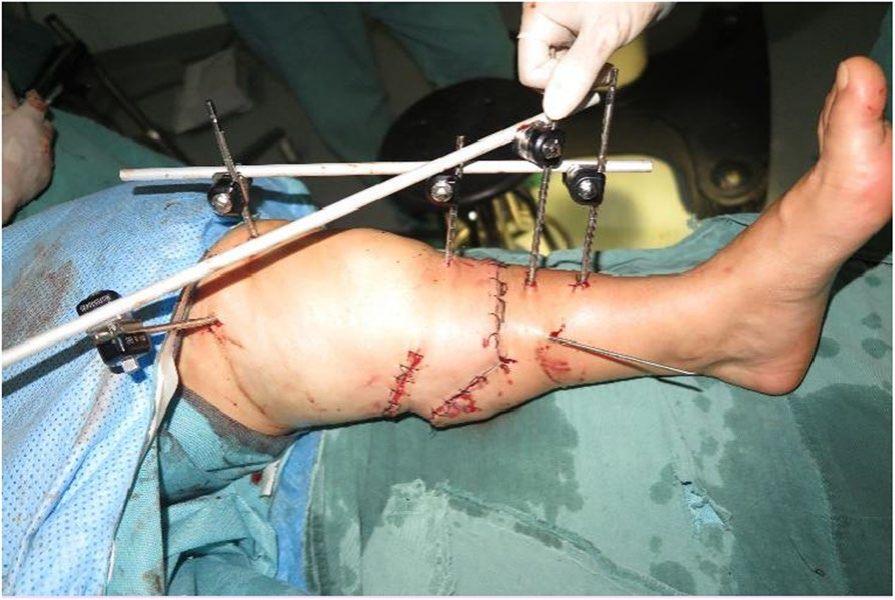
Skin closed and external fixation

We microscopically examined viable vasculature and nerves for reconstruction. We extracted and repaired the popliteal artery, anterior tibial artery, and accompanying veins, he great saphenous vein, the tibial nerve, and the distal length of the common peroneal nerve. Distorted tendons were repaired with tendon suture. After loosening the thigh tourniquet to confirm a healthy blood supply to the foot and normal capillary refill time, the skin was closed, and external fixation was reinforced for bone strength and stability (Fig. 7).

Due to extensive tissue damage, a portion of the wound on the posterolateral leg was intentionally left open to heal secondarily. The patient required another procedure 3 months post-operative to address the remaining recalcitrant wound, which was treated with skin grafting.

**Fig. 8:**
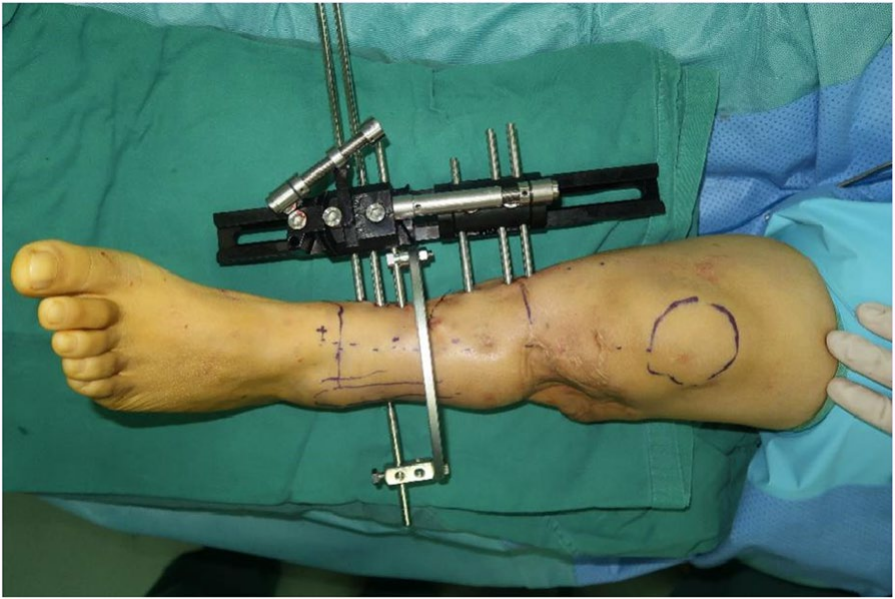
Osteotomy and limb lengthening procedure with external fixation

One year after the primary surgery, the patient returned to the hospital for further treatment due to a residual leg shortening deformity, resulting in a 15 cm difference in length compared to the unaffected leg. This shortening significantly affected her daily activities. The patient was scheduled and underwent an osteotomy and limb lengthening procedure with external fixation (Fig. 8).

## Results

Out of 12 patients, 10 were males (90%) and 2 were females (20%) (Table 1). The median age was 44 years (range 20–60 years). The majority of injuries were related to motor vehicle accidents (eleven out of 12 cases). Ten patients (90%) underwent successful secondary surgeries to correct limb shortening or deformities. All amputations occurred below the knee – right limb amputation (*n* = 8) and left limb amputation (*n* = 4). Eleven patients were employed at the time of the interview, and one was retired. The median time from primary surgery to interview was 15 months (range 7–45 months) (Table 1).

**Table 1:** Interview Information of Patients

Sex/age (years)	Cause of injury	Diagnosis	Primary/ Secondary Surgeries	Major complications	FU: surgery-interview (months)
F/20	MVA	RLEA	Replantation: Bone lengthening	Limb shortening deformity	18
M/55	MVA	RLEA	Replantation: Bone lengthening	Limb shortening deformity	7
F/37	MVA	RLEA	Replantation: Bone lengthening	Limb shortening deformity	15
M/43	MVA	RLEA	Replantation: Bone lengthening	Limb shortening deformity	12
M/47	MVA	LLEA	Replantation: Bone lengthening	Limb shortening deformity	34
M/34	WRI	RLEA	Replantation	Contractures	29
M/27	MVA	LLEA	Replantation: Bone lengthening	Limb shortening deformity	9
M/46	MVA	LLEA	Replantation	Scaring	13
M/47	MVA	RLEA	Replantation: Bone lengthening	Limb shortening deformity	8
M/56	MVA	RLEA	Replantation: Bone lengthening	Limb shortening deformity	24
M/49	MVA	LLEA	Replantation: Bone lengthening	Limb shortening deformity	20
M/38	MVA	RLEA	Replantation	Contractures	27

### Theme 1: Satisfaction with quality of life despite activity of daily living limitations

Although 10 out of 12 candidates showed concern about their inability to engage in active physical activities as compared to before the injury, they were satisfied with the eventual outcomes despite the limitations in physical activities such as running, jogging and hiking. The patients were satisfied with the independence in carrying out their activities of daily living despite having to improvise for certain higher-level functions and exercise. (“staying physically active has been my life goal and I sometimes feel life I have failed, but I am glad I could still walk on my own legs and stay active. I am always happy after exercise, you know?”- A 34-year-old male interviewed at 16 months after limb shortening correction). All patients expressed satisfaction with their choice of replantation procedure as compared to amputation and subsequent prosthetic limb fitting. (“going through two surgeries and the financial burden associated with it was tough for me and my family, but I have my leg back. I wonder how I would live with part of my body missing. The scars look ugly, I know, but I love it more than a beautiful foreign material attach to my body.”- A 47-year-old female interviewed twelve months after her primary surgery). All patients acknowledged the functional limitations from their traumatic injury but appreciated the independence and being in control of their lives. (“I don’t feel comfortable wearing shorts as I used to do during summer but who cares? I still have my job and I can take of my family. That’s all that matters, right?” -A 37-year-old male interviewed 15 months after the primary surgery) (Table 2).

**Table 2:** Additional quotes organized by theme from participants

Themes & Participants	Quotes
**Reluctance to accept alternative treatment**	
A 20-year-old female, 18m after limb lengthening surgery.	‘’this might sound crazy, but I will rather die than to live with one leg or some plastic attached to my body’’. I know life is important; I just can’t.
A 47-year-old female, 34mths after secondary surgery.	“This can happen to anyone, it’s terrible experience; I prefer to leave the world the way I came in” hmm! I’m happy have my imperfect leg back.”
A 34-year-old male, 29m after replantation procedure.	“I know my family would support and love me with or without my leg, but I don’t think I can face life with anything that’s not my own body part; I just wanted my leg fixed even if it won’t be perfect.”
A 49-year-old male, 20m after limb lengthening surgery.	“The worst part of all this when you know there a chance to get your life back; it can’t be perfect, but at least I won’t feel so terrible about myself.”
**Sense of purpose and social engagement**	
A 38-year-old male, 27m after primary surgery.	“Sometimes you feel horrible about how your body has changed…but I am still useful and able to do what I love.” Life must go on because you have a family and work to keep “you live everyday…. you know, it makes you think less about your appearance.”
A 58-year-old male, 24m after limb lengthening surgery.	“Hmmm! I’ve been leading evening dance group and I loved it- I can’t assume that role anymore, but I can still enjoy dancing with my team.”

### Theme 2: Social support network influences on satisfaction

All patients interviewed were grateful for the support they received from their immediate family members. There was also deep appreciation for the doctors and other caregivers who provided medical care during their recovery and rehabilitative phases. (“I couldn’t have survived this without my wife’s constant support.” A 46-year-old male interviewed 13 months after the replantation surgery).

Patients were also grateful for the support received from their social networks. (“it hurts when I couldn’t play basketball anymore with my friends, the truth is, I never thought I could live without playing basketball for the rest of my life. I am thankful that I can still play badminton with my friends. In fact, I don’t feel like missing anything, life is always good with friends- trust me.” A 27-year-old male interviewed 9 months after the bone lengthening surgery).

One of the patients, who was gainfully employed, divorced, and did not have any strong social ties expressed frustration and struggled to come to terms with his new life, in contrast with patients who had strong familial and social support. (“it’s hard when life is so unfair to you, and you don’t have your family around. I loved playing soccer with my friends before my accident, but I felt they abandoned me because I couldn’t play with them any longer. I am glad to be alive, of course, but I wished all these never happened.” – A 56-year-old male interviewed 24 months after his incident).

### Theme 3: Social contribution, sense of purpose and finding meaning

11 out of 12 patients who were employed at the time of the interview were more satisfied after recovery due to the positive influences of social participation. (“I was scared to death that I will lose my contribution to the society. Being able to walk on my two feet and go to work every day gave the reason to live. I wake up every day feeling happy to dress up to work. I don’t really miss a whole lot.”- A 38-year-old man interviewed 27 months following the limb replantation procedure).

The patient who was retired at the time of interview expressed dissatisfaction and impaired sense of purpose. (“I retired earlier than I should have because I didn’t feel able enough to continue working after my injury. I’ve always loved to work, but I had to let all that go earlier than I should all because of this crazy accident. Believe me, it’s not fun when you have nothing to doing all day. I am useless.” – A 55-year-old male interviewed 18 months after surgery).

### Theme 4: Acceptance and life satisfaction despite cosmetic deficiencies

9 out of 12 patients interviewed expressed concern relating to body image issues from the resultant scars and residual deformities. However, they preferred the complications to living with a missing limb. (“I have always cared about my body, and I didn’t have a single scar on my body before my accident. Now, this huge scar on my leg looks ugly but am glad I still have my own leg instead of losing it or having an artificial leg. I can’t accept to live like that, you know. I feel complete again.” – A 37-year-old female interviewed 20 months after her surgery).

(“part of my body can’t be gone while am still alive. I was born with my leg, and I must go back with it. Can you accept this this? Well, I can’t. I thank the doctors for fixing it.” – A 44-year-old male interviewed 30 months after surgery)

## Discussion

Lower limb amputation is life-changing changing event and the impact on quality of life can be debilitating for many years [6–9]. After a lower-leg amputation or amputation-like injury, treatment options include stump provision, early prosthesis fitting, or replantation.

Qualitative analysis of interviews with 12 patients who underwent lower limb replantation and subsequent limb lengthening surgeries demonstrated overall life satisfaction, despite the associated complications and multiple surgeries. Surgical interventions are often evaluated based on the physician’s perspective of success. However, it is equally important to assess outcomes from the patient’s perspective and to consider the patient’s quality of life beyond replanted limb and life preservation. When assessing the outcomes of life-changing trauma and procedures, it is essential to view things from the patient’s perspective. Are the patients satisfied with the procedures the underwent, or are they merely surviving?

In this study, the authors analysis of the interviews with patients who underwent limb replantation and lengthening surgeries demonstrated satisfaction with their outcomes and overall health. While this finding may be considered intuitive, it is relevant in a society where changes in body image are often met with disapproval.

Although the replantation procedures performed in this series were surgically possible, and the limb lengthening surgeries were indicated, other factors such as psychological considerations, patient’s attitudes, characteristics, and values play a significant role in decision-making process regarding replantation procedures, going beyond surgical feasibility and indications.

The benefits of primary amputation include earlier discharge, functional recovery with prosthesis fitting, and social integration [10–12]. Furthermore, if a patient were to require subsequent delayed amputation due to a failed replantation procedure, the morbidity, mortality, and financial impact on patients are significantly increased as compared to patients who undergo a primary amputation. [10] Compared to limb replantation, the multiple procedures, extended hospital stays, and prolonged recovery can significantly impact patient’s satisfaction, as supported by multiple previous studies [13, 14].

However, primary amputation and prosthetic applications are associated with significant complications, including gait distortions, skin maceration, ulceration, and chronic pain [11]. Reduced physical ability to perform manual labor and acceptance of a prosthesis can significantly affect a patient’s physical and psychological recovery.

In the current study, all patients expressed satisfaction with their quality of life and overall health. Patient satisfaction evaluations are closely linked to the success of surgical interventions.

In patients with life-threatening traumatic limb amputations, primary amputation is indicated to preserve the patient’s life. However, limb replantation surgery may be considered as an option, depending on the patient’s physiology, soft tissue quality, and bone condition, provided that suitable surgical expertise is available [15, 16]. In our experience, the success of replantation is significantly influenced by the experience of the surgical team. However, patient satisfaction after surgery may be more important than successful surgical outcome, and this is significantly influence by sociodemographic factors, body image perception, and patient’s underlying attitudes towards prosthetic limb fitting.

Despite the reported downsides, replantation offers justifiable advantages compared to primary amputation. With careful patient selection and availability of expertise, a functional extremity can be attained in 80-90% of case [17, 18], which provides numerous benefits over a traditional lower limb prosthesis. In this series, all patients achieved functional independence following limb lengthening operations.

Body image perception and associated psychological problems may vary among cultures. All patients interviewed expressed that the loss of a body part was culturally unacceptable in their respective cultures. These cultural views on the loss of a body part may explain the level of satisfaction expressed during the interview.

Our research has some limitations. Qualitative research methodologies are not designed to produce entirely generalizable conclusions; instead, they are employed to obtain in-depth and contextual knowledge of the participants’ experiences. It worth noting that the experience of patient’s family members and caregivers were not recorded. While inclusion of family and caregivers in the interviews could provide additional insights, they were intentionally excluded as part of the research strategy. This approach was chosen to allow a focused understanding of the patients’ views without undue influence.

## Conclusion

Despite the risks of delayed amputation, greater financial expenditures, and longer recovery time associated with lower-limb replantation compared to primary amputation and early prosthetic fitting; replantation should be discussed as an option with patients if it is surgically indicated. Patients who undergo lower limb replantation can experience significantly improved quality of life, especially when there is strong social support, the ability to contribute in a meaningfully to their communities after surgery, and acceptance of any cosmetic deficiencies.

## Data Availability

The datasets used and/or analysed during the current study available from the corresponding author on reasonable request.
